# Artemisinin combination therapy mass drug administration in a setting of low malaria endemicity: programmatic coverage and adherence during an observational study in Zanzibar

**DOI:** 10.1186/s12936-017-1982-x

**Published:** 2017-08-14

**Authors:** Abdullah S. Ali, Narjis G. Thawer, Bakar Khatib, Haji H. Amier, Joseph Shija, Mwinyi Msellem, Abdul-wahid Al-mafazy, Issa A. Garimo, Humphrey Mkali, Mahdi M. Ramsan, Jessica M. Kafuko, Lynn A. Paxton, Richard Reithinger, Jeremiah M. Ngondi

**Affiliations:** 1 Zanzibar Malaria Elimination Programme, Ministry of Health, Zanzibar, Tanzania; 2 RTI International, Dar es Salaam, Tanzania; 3 President’s Malaria Initiative/United States Agency for International Development, Abuja, Nigeria; 4 President’s Malaria Initiative/Centers for Disease Control and Prevention, Dar es Salaam, Tanzania; 5 RTI International, Washington, DC USA

**Keywords:** Mass drug administration, Zanzibar, Artemisinin-based combination therapy, Hotspots, Adherence

## Abstract

**Background:**

Mass drug administration (MDA) appears to be effective in reducing the risk of malaria parasitaemia. This study reports on programmatic coverage and compliance of MDA using artemisinin-based combination therapy (ACT) in four *shehias* (smallest administration unit) that had been identified as hotspots through Zanzibar’s malaria case notification surveillance system.

**Methods:**

Mass drug administration was done in four *shehias* selected on the basis of: being an established malaria hot spot; having had mass screening and treatment (MSaT) 2–6 weeks previously; and exceeding the epidemic alert threshold of 5 cases within a week even after MSaT. Communities were sensitized and MDA was conducted using a house-to-house approach. All household members, except pregnant women and children aged less than 2 months, were provided with ACT medicine. Two weeks after the MDA campaign, a survey was undertaken to investigate completion of ACT doses.

**Results:**

A total of 8816 [97.1% of eligible; 95% confidence interval (CI) 96.8–97.5] people received ACT. During post MDA surveys, 2009 people were interviewed: 90.2% reported having completed MDA doses; 1.9% started treatment but did not complete dosage; 4.7% did not take treatment; 2.0% were absent during MDA and 1.2% were ineligible (i.e. infants <2 months and pregnant women). Main reasons for failure to complete treatment were experience of side-effects and forgetting to take subsequent doses. Failure to take treatment was mainly due to fear of side-effects, reluctance due to lack of malaria symptoms and caregivers forgetting to give medication to children.

**Conclusion:**

Mass drug administration for malaria was well accepted by communities at high risk of malaria in Zanzibar, with high participation and completion rates. Further work to investigate the potential of MDA in accelerating Zanzibar’s efforts towards malaria elimination should be pursued.

## Background

Malaria was the leading cause of morbidity and mortality in Zanzibar prior to 2003, with malaria endemicity being classified as moderate to high (i.e. parasitaemia among symptomatic patients tested was 35–40%) [1]. In response to the malaria burden, the Zanzibar Malaria Control Programme—in collaboration with bilateral, multilateral and non-governmental partners—scaled up prevention and control efforts, including: artemisinin-based combination therapy (ACT); insecticide-treated nets (ITNs); indoor residual spraying of households with insecticide (IRS); and intermittent preventive treatment for pregnant women (IPTp) with sulfadoxine–pyrimethamine (SP) [2–4]. Over the past decade these efforts changed malaria endemicity in Zanzibar from hyper- to hypo-endemic, with parasite prevalence dropping to and being maintained at <1% for the previous 3 years [1, 4].

Building on the success of these efforts and in alignment with global guidance [5], Zanzibar is now targeting malaria elimination on both Unguja and Pemba islands: the goal is to achieve zero locally acquired cases by 2018 by achieving and maintaining 100% coverage with appropriate prevention measures [6]. While malaria elimination relies upon a similar mix of interventions as during the malaria control phase (i.e. case management, vector control, and surveillance), it requires more intensified, rapid, and targeted responses, especially for targeting transmission hotspots and specific high-risk populations [7–9]. The Zanzibar Malaria Elimination Programme (ZAMEP) is able to identify hotspots through its malaria case notification (MCN) (“Coconut”) surveillance system, which tracks daily facility-based malaria cases for real-time decision-making and active case detection through household screening and treatment (HSaT).

One potential strategy for targeted interventions in a malaria elimination setting is focal mass drug administration (MDA) in high-risk populations. Although falling out of favour due to efficacy concerns during the eradication era in the 1960s, MDA has re-emerged as a potential strategy for malaria elimination and eradication [10, 11]. Use of MDA since the eradication era has mainly been preventative therapy and seasonal chemoprevention; however, an argument for MDA is that diagnosis can be hampered by lack of biomarkers of the liver stage of malaria and limitations of currently available detection methods for lower density blood stage infections [12, 13]. A recent systematic literature review found that: (1) MDA has been used in large-scale malaria control, elimination and outbreak response efforts, with community participation and at least 80% coverage of the target population being important factors for a successful MDA campaign; (2) MDA and mass chemoprophylaxis probably contributed to reducing parasite prevalence in certain intervention settings; and (3) select MDA programmes have been considered successful based on qualitative assessments other than efficacy [14]. Renewed interest in MDA as an approach for interrupting transmission is based on the availability of effective anti-malarial drugs with transmission reducing characteristics as well as mathematic modeling studies showing the epidemiological impact of the intervention in settings with low transmission intensity and during periods of low vector densities [15]. However, there is evidence to suggest that effectiveness of MDA is often not sustained beyond 6 months [16].

Taking into account the current pre-elimination setting of Zanzibar, MDA might have a significant effect when used focally in areas at risk of high-transmission, especially before the peak malaria transmission season. This study reports on programmatic coverage and compliance of MDA using ACT in four *shehias* (the smallest administrative unit in Zanzibar comprising of 2000–5000 population) that had been identified as hotspots through Zanzibar’s MCN surveillance system.

## Methods

### Study settings

**Fig. 1:**
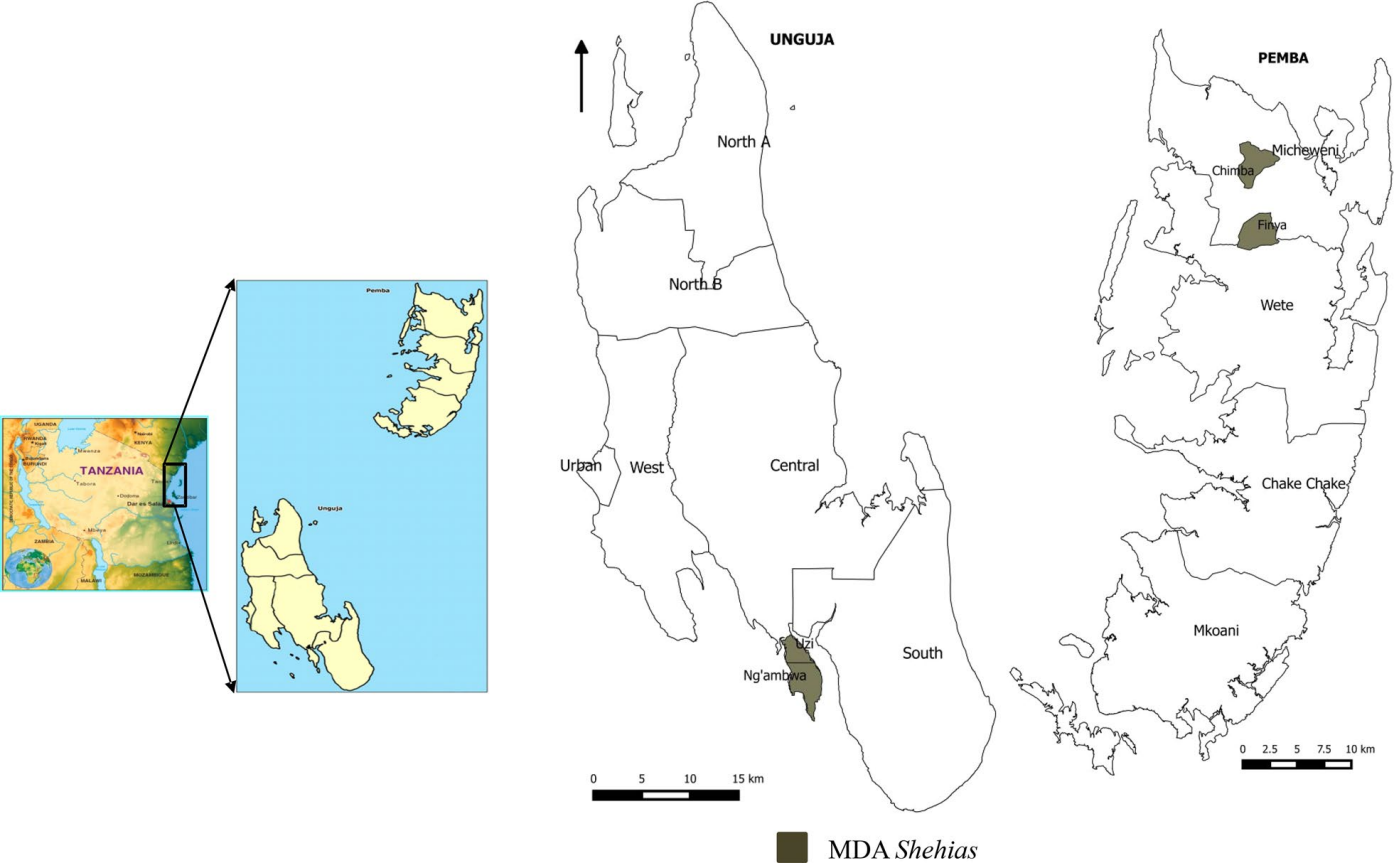
Map of Zanzibar showing the MDA *shehias*

There are 331 *shehias* in Zanzibar, of which four were purposively selected for the MDA intervention, because they: (1) were established malaria transmission hotspots with annual malaria incidence >20/1000 population; (2) had received mass screening and treatment (MSaT) 2–6 weeks earlier; and (3) had exceeded the epidemic alert threshold of 5 cases or more reported within a week even after MSaT intervention, with MDA considered as an additional intervention [17]. Figure 1 shows the location of the *shehias* where the MDA was undertaken in June 2013: Finya and Chimba are located in Micheweni District of Pemba, while Uzi and Ng’ambwa is a peninsula in the South District of Unguja.

### MDA implementation

#### Drug used for MDA

Table 1 shows the ACT preparations and dosages used during the MDA. The main ACT used was dihydroartemisinin/piperaquine phosphate (Duo-Cotecxin^®^, Beijing Holley-Cotec Pharmaceutical Co Ltd, China). In Uzi and Ng’ambwa, artemisinin and piperaquine (Artequick^®^, Artepharm, China) was also dispensed due to shortages of Duo-Cotecxin. Duo-cotecxin and Artequick^®^ were considered appropriate for the MDA pilot, because both are not used in the routine treatment of malaria in Zanzibar. However, based on the manufacturers recommendations, Duo-Cotecxin^®^ 40/320 mg tablets is not suitable for children aged <6 months while Artequick^®^ 62.5/375 mg tablets is not suitable for children age <7 years [18, 19]; therefore, artesunate–amodiaquine (ASAQ) Winthrop^®^ was dispensed instead.

**Table 1:** List of ACT preparations and dosages used during the MDA

ACT drug	Formulation	Age group	Daily dosage (tablets)	Number of days
Dihydroartemisinin and Piperaquine Phosphate (Duo-Cotecxin^®^)	40/320 mg tablets	6 months–1 year	½	3
		2–7 years	1	3
		8–13 years	2	3
		≥14 years	3	3
Artemisinin and Piperaquine (Artequick^®^)^a^	62.5/375 mg tablets	7–10 years	1	2
		11–15 years	1½	2
		≥16 years	2	2
Artesunate Amodiaquine Winthrop^®b^	25/67.5 mg tablets	2–11 months	1	3
	50/135 mg tablets	1–5 years	1	3

^a^Used in Uzi and Ng’ambwa only

^b^Dispensed for children not eligible for Duo-Cotecxin^®^ 40/320 mg tablets (age <6 months) and Artequick^®^ 62.5/375 mg tablets (age <7 years) [18, 19]

#### Population eligible for MDA

All people living in the MDA pilot areas were eligible for treatment, except infants <2 months of age, and pregnant women in the first trimester of pregnancy.

#### Training

In each *shehia*, ten teams comprising of a healthcare worker (i.e. drug dispenser), an administration leader (i.e. local guide) and a recorder (to complete MDA monitoring forms) were trained to conduct MDA. In addition to the drug distribution team, a team comprising a health promotion specialist and a local guide were allocated to each *shehia* to conduct community mobilization and top-up ITN distribution on the day of the MDA. A days’ training was conducted to drug distribution teams with the objective of explaining the rationale and importance of MDA, filling MDA forms and dispensing of ACT.

#### Community mobilization

Two days prior to MDA implementation, a series of community mobilization events were undertaken in each *shehia*, including radio programmes on malaria prevention and response activities, house-to-house communication through local leaders, and public announcements through town criers. The information focused on what, why, when and how MDA would be carried out. Information leaflets on the MDA activity and malaria prevention in Swahili language were also distributed in the target *shehias*. After the MDA, public announcements continued on the second and the third day with messages targeted at reminding communities to take the second and the thirds ACT doses. Information on malaria prevention through consistent use of bed nets was also delivered.

#### Drug distribution

The MDA was undertaken from house-to-house. In each household, all members were enumerated. Women of child-bearing age were asked if they were in the first trimester of pregnancy to determine eligibility for treatment. All eligible persons present were offered ACT, with the first dose being taken as a directly observed treatment (DOT). Dosage for subsequent days (day 2 and 3) was dispensed in packets labeled with the name for each eligible recipient. For those that were not present during the household visit, drugs were dispensed in a labeled packet and left with the head of the household. Instructions on when the subsequent doses would be taken were provided to all MDA adult participants. Parents and guardians were provided with information on dosing of children. After the MDA, public announcements were done in the community on days 2 and 3 post-MDA reminding participants to take the dose for the respective day.

### Post MDA survey

Two weeks after the MDA, a survey was done to estimate completion of ACT doses distributed; investigate reasons for not completing MDA doses; investigate side-effects following MDA; and explore willingness of participants to take part in MDAs in the future.

Using an internet-based sample size calculator (http://www.raosoft.com/samplesize.html), sample size was calculated to estimate 50% completion of all three MDA doses within a 5% error margin, given a 5% level of significance and 95% confidence level. Calculations were adjusted for a design effect of 2.0 to allow for household clustering of observations, as well as a 10% non-response rate, yielding a minimum sample size of 750 individuals required per *shehia*. Assuming—based on operational programme implementation experience—a household size of 5, at least 150 households were required to be surveyed in each *shehia*. A list of all households that would be eligible to participate in the MDA was obtained for each *shehia*. To ensure the sample’s uniform distribution across the *shehia*, household lists were ordered by *sub*-*shehia* prior to 150 households being selected using systematic random sampling.

### Data collection and analysis

Customized forms were designed to collect data during the MDA distribution and post-MDA survey. MDA monitoring forms included a household census, eligibility for participation in MDA and ACT dosage. Post MDA forms included a household census, questions on MDA participation, reasons for MDA non-completion, side-effects and willingness to participate in MDA in the future. Data were completed by health care workers who were experienced in undertaking household surveys and who had received a day’s training on the survey protocol. Data from the MDA monitoring form and post-MDA survey were entered using Microsoft Excel^®^ (Microsoft Corporation, Redmond, WA) spreadsheets. Statistical analysis was conducted using Stata 12.0 (Stata Corporation, College Station, TX). Data for Uzi and Ng’ambwa were combined together for analysis, because the two *shehia* comprise a peninsula on Unguja. Descriptive statistics were used to summarize the age and gender distribution of the MDA populations and post-MDA sample, MDA treatment coverage and MDA completion. Differences in proportions were compared using the χ^2^ test.

## Results

### Age and gender characteristics of study population

A total of 9076 participants in 2001 households were enumerated in the four districts targeted for MDA; a total of 2009 participants were enumerated in 413 households during the post MDA survey. Compared to Chimba and Finya, the mean age of the population of Uzi and Ng’ambwa was slightly higher; however, there was no statistically significant difference in the gender distribution across *shehias* (Table 2).

**Table 2:** Age and gender characteristics of population participating in MDA and sample of post MDA participants

Population	Number of households	Number of people enumerated	Mean age (SD) years	Male Gender (%)
Population participated in MDA				
Chimba	672	3399	20.6 (17.2)	50.3
Finya	471	2495	20.8 (17.5)	49.5
Uzi & Ng’ambwa	858	3182	22.8 (17.8)	51.5
Total	2001	9076	21.4 (17.5)	50.5
Sample participated in post MDA survey				
Chimba	150	746	19.9 (17.2)	49.3
Finya	150	770	20.7 (17.0)	50.6
Uzi & Ng’ambwa	113	493	22.4 (17.5)	51.9
Total	413	2009	20.9 (17.3)	50.5

*SD* standard deviation

### MDA treatment coverage

**Fig. 2:**
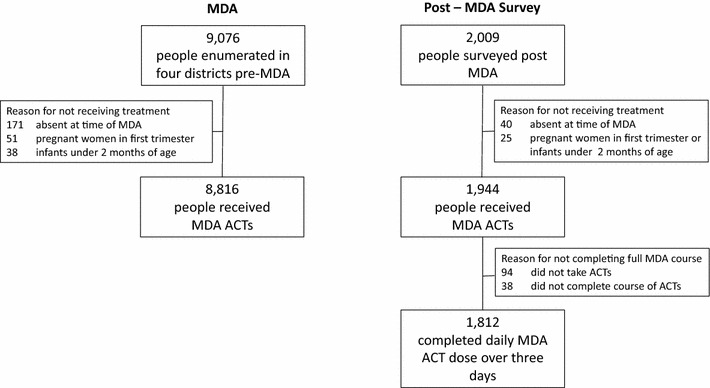
Consort chart of study

Overall, a total of 8816 out 9076 [97.1%; 95% confidence interval (CI) 96.8–97.5] people enumerated received treatment, with MDA coverage between districts not significantly different, ranging from 95.7%; (95% CI 94.0–96.4) in Uzi and Ng’ambwa to 98.0% (95% CI 97.4–98.4) in Chimba. Of the 260 people that did not receive treatment, the majority (65.8%) were absent from the household at the time of the MDA; the remainder were not eligible to receive treatment [i.e. infants under 2 months of age (14.6%) and pregnant women in the first trimester (19.6%)] (Table 3; Fig. 2).

**Table 3:** MDA treatment coverage by *shehia*

*Shehia*	People enumerated	People treated	Proportion treated % (95% CI)
Chimba	3399	3331	98.0 (97.5–98.4)
Finya	2495	2439	97.8 (97.1–98.3)
Uzi & Ng’ambwa	3182	3046	95.7 (94.9–96.4)
Total	9076	8816	97.1 (96.8–97.5)

*CI* confidence interval

### MDA treatment completion

A sample of 2009 people were interviewed during post-MDA survey, of whom: 90.2% reported having completed MDA doses; 1.9% started treatment but did not complete dosage; 4.7% received medicine but did not take treatment; 2.0% did not receive medicine, because they were absent during MDA; and 1.2% were not eligible to receive treatment (infants and first trimester pregnant women) (Table 4; Fig. 2). Of the 38 people not completing treatment, two fifths did not provide any reason; the remainder provided a range of reasons for non-compliance, including experienced side-effects, fear of side-effects, and forgetting to take treatment. Reasons for failure to take treatment were: fear of side-effects; did not have fever; forgot to take treatment or forgot give treatment to children; fasting; busy with daily chores; and insufficient information about MDA. A total of 94 (i.e. 5.2% of those taking treatment) reported side-effects including: muscular aches (22.3%); dizziness (21.3%); abdominal pain (16.0%); vomiting (11.7%); headache (9.6%); fever (5.3%); sneezing (4.3%); diarrhoea (3.2%); nausea (3.2%); other (3.2%). The majority (94.2%) of 2009 survey participants reported that they would take part in MDA in the future.

**Table 4:** MDA completion of ACT doses in Chimba, Finya, Uzi and Ng’ambwa *shehias*

*Shehia*	Survey participants	Participation in MDA (%)				
		Completed MDA dose	Started treatment but MDA dose not completed	Refused to take MDA	Not present during MDA	Not eligible for MDA
Chimba	746	89.4	2.9	5.4	1.2	1.1
Finya	770	91.3	1.0	4.5	1.8	1.3
Uzi and Ng’ambwa	493	89.7	1.6	3.9	3.7	1.2
Total	2009	90.2	1.9	4.7	2.0	1.2

## Discussion

This study presents findings on the community’s acceptability, completion and perceptions after focal MDA with ACT intended to mitigate increased seasonal malaria transmission in Zanzibar. MDA was conducted within communities as a response to an increase in malaria cases in the four *shehias* defined as hotspots where MDA was recommended because—although MSaT had been done a few weeks prior—the number of malaria cases in these *shehias* continued to increase. The rationale for MDA was to provide appropriate treatment to clear malaria parasites in the population so as to prevent on-going transmission in malaria hotspots with intense transmission. The findings of the pilot presented here showed high MDA participation (97.1%) and acceptance by the communities targeted, with high completion of ACT doses (90.2%) among participants who received the medication. This finding was most likely attributed to the intensive community sensitization and mobilization activities that took place prior to the MDA, including the house-to-house approach as well as the post-MDA public announcements to remind people to complete subsequent doses. The findings also suggest that community sensitization was effective, as the majority (94.2%) of survey participants reporting that they would take part in future MDAs. This suggests that MDA can be considered as a practical intervention in persisting malaria hotspots, particularly if active case detection efforts such as MSaT and HSaT fail to adequately curb transmission.

The post-MDA survey findings showed that the reasons for failure to complete the ACT doses were fear of side-effects such as abdominal pain, headaches, vomiting, dizziness, sneezing, muscular aches, fever, nausea and body malaise. Similar findings were observed in studies conducted in The Gambia where barriers of the MDA campaign included the perception and fear of the community members on the side-effects of the drugs and procedures [20, 21]. These misconceptions by the participants and misunderstandings about the medication can contribute greatly to the lack of adherence to an MDA [22]. Similarly, fear of side-effects from the drugs leading to decreased MDA coverage has also been reported in MDA for other parasitic diseases, e.g. lymphatic filariasis [23–27]. Other reasons for non-compliance reported by participants in Zanzibar included: forgetting to take treatment; insufficient information about MDA; reluctance to take drugs without malaria symptoms; fasting; and busy with daily chores. The number of pills required for a full ACT regimen has also been shown to discourage adherence to the MDA programme [28]. Single dose treatment, possibly under DOT, could enhance compliance for malaria MDA.

As neglected tropical disease MDAs have shown in multiple countries [29–31], in order to maximize MDA participation, misconception and fears of side-effects within the target population need to be addressed at the start of the activity and frequent communication should be maintained throughout the MDA period through community education and training of health care workers [14, 26, 32]. This is particularly salient if several rounds of MDA were to be conducted in order to have a more lasting impact on malaria transmission. In addition, community sensitization should involve village leaders to enhance participation from community members [20]. The importance of educating the target community with correct information regarding the drug, procedures to be taken and the possible side-effects helps to enhance adherence. The results of this study show that MDA is a practical response intervention in low transmission settings; however, further research is needed to investigate the effectiveness of MDA in the four *shehias*.

This is the first study to pilot MDA as an intervention for malaria elimination in Zanzibar. The study has several potential limitations. Firstly, the pilot was done in a few *shehias* selected purposively and, therefore, may not be generalizable to the whole of Zanzibar. Secondly, compliance for treatment was measured based on self-reports by the participants, which may be subject to recall bias or over-reporting. Finally, the study was designed as a feasibility study embedded in a larger operational program, and not as an efficacy study to measure the impact of MDA on malaria morbidity and/or transmission. If MDA does become part of the intervention package to ensure malaria transmission remains low, particularly in hotspots, then a robust monitoring and evaluation framework and protocol to measure the intervention’s efficacy will have to be designed.

## Conclusion

This study demonstrates that MDA for malaria was feasible and highly accepted by communities at high risk of malaria in Zanzibar, with high participation and completion rates, even though they had recently been targeted for MSaT. Together with other preventative measures, MDA has the potential to contribute to interrupting transmission of malaria and accelerate ZAMEP’s efforts towards malaria elimination.

## Abbreviations

ACT: artemisinin-based combination therapy
MDA: mass drug administration
